# A case of afterload mismatch associated with shivering leading to fatal hypoxia in a COVID-19 patient

**DOI:** 10.1186/s40981-022-00542-3

**Published:** 2022-07-16

**Authors:** Takanori Suzuka, Yusuke Naito, Keiko Uemura, Mitsuru Ida, Junji Egawa, Masahiko Kawaguchi

**Affiliations:** Department of Anesthesiology, Nara Medical University, Kashihara, Japan

**Keywords:** COVID-19, Shivering, Afterload mismatch

## Abstract

**Background:**

Fever and associated shivering are frequent symptoms in patients with coronavirus disease 2019 (COVID-19). High body temperature activates the immune system, which might be beneficial. However, shivering leads to high oxygen demand.

**Case presentation:**

A 38-year-old man diagnosed with COVID-19 was transferred to our intensive care unit (ICU). His oxygen saturation (SpO2) level was approximately 92–95% and was managed with a high flow nasal cannula. Six hours after admission to the ICU, he started shivering, and his systolic blood pressure rose above 200 mmHg. Concomitantly, his SpO2 levels decreased rapidly. Mechanical ventilation was started, but oxygenation could not be maintained, requiring the establishment of extracorporeal membrane oxygenation (ECMO).

**Conclusions:**

COVID-19 is known to cause thrombosis in the pulmonary microvasculature at the early stage of the disease. Under these circumstances, caution should be paid since shivering may worsen the patient’s condition.

## Background

Fever is a cardinal symptom of coronavirus disease 2019 (COVID-19), which is observed in approximately 10% of patients. Shivering is an important physiological function for heat production, which is achieved by the elevation of plasma adrenocorticotropic hormone and catecholamines. However, the release of such hormones leads to various adverse effects, such as increased blood pressure and systemic vascular resistance (SVR), which negatively affect cardiopulmonary function [1]. Microthrombi have been reported to form in the pulmonary arteries of patients with COVID-19 at the early stages of the disease. As a result, regions of the pulmonary vasculature without microthrombi experience both increased pressure and increased blood flow, leading to increased pulmonary capillary hydrostatic pressure and subsequent leakage of fluid into the interstitial and alveolar spaces. This phenomenon, called over-perfusion edema, initially occurs in a non-injurious, dynamic manner, with quickly reversible changes in the alveolar capillary permeability. However, if the hydrostatic pressure rises sufficiently, mechanical rupture with capillary stress failure occurs [2].

Afterload mismatch is a condition in which the heart with diastolic dysfunction is unable to absorb the rapid increase in the left ventricular end-diastolic volume. This is caused by stresses, such as exercise, rapid fluid infusion, or increased sympathetic nervous system reaction, resulting in an increase in the left ventricular end-diastolic pressure [3].

In this report, we describe a case of a male patient with COVID-19 who developed pulmonary edema, presumably due to an afterload mismatch triggered by shivering, resulting in rapid progressive fatal hypoxia requiring extracorporeal membrane oxygenation (ECMO).

## Case presentation

**Fig. 1:**
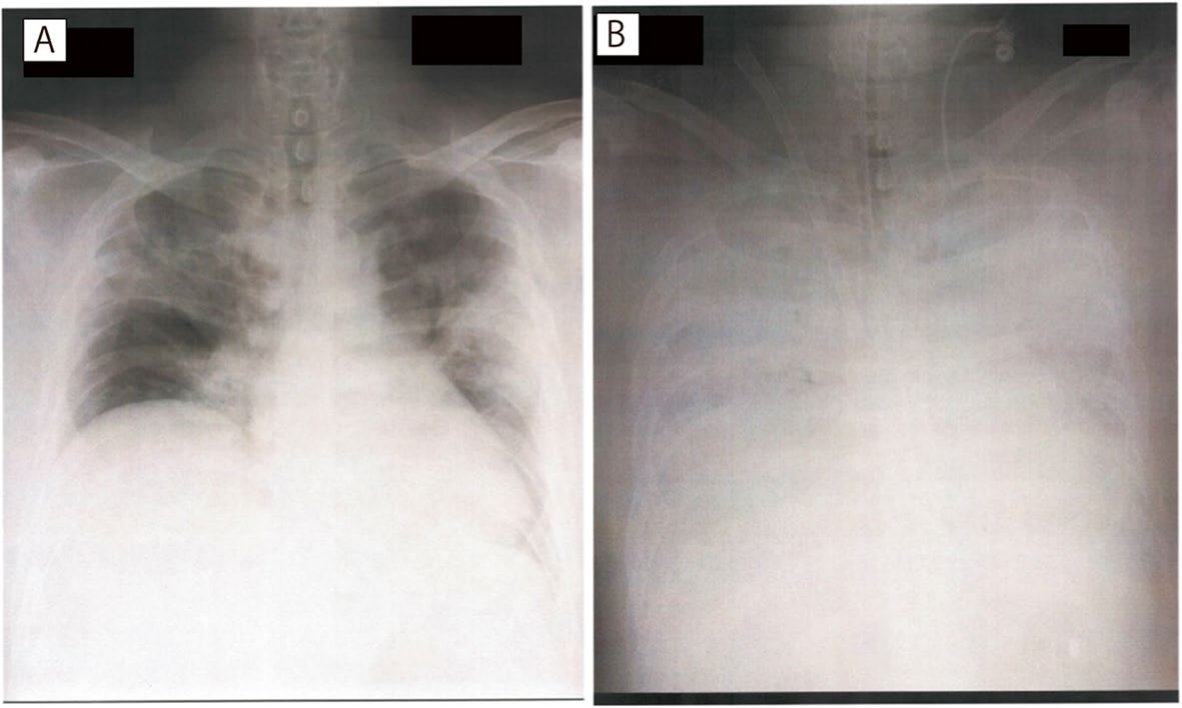
Chest radiograph on admission to the ICU (**A**) and immediately following the initiation of extracorporeal membrane oxygenation (ECMO) (**B**). Diffuse shadows in the bilateral peripheral lung on admission and enhanced pulmonary vascular shadow with cardiac enlargement and decreased permeability after starting ECMO should be noted

Written informed consent was obtained from the patient for the publication of this case report and accompanying images. A 38-year-old male patient, who was 165-cm tall and weighed 110.3 kg (body mass index, 41 kg/m^2^), reported a history of type 2 diabetes with medical control and no history of cardiac failure, with normal exercise tolerance. He visited another hospital with a chief complaint of fever and was diagnosed with COVID-19 through reverse transcription-polymerase chain reaction. No computed tomography (CT) scan was performed before admission into the intensive care unit (ICU). On the same day, he was admitted to the ICU in our hospital (i.e., on the 7th day after the onset of symptoms) as his oxygen saturation (SpO_2_) level was around 85% under a reservoir mask of 8 L/min on exertion, and multidisciplinary treatment was deemed necessary. On admission to the ICU, SpO_2_ level was 94% (reservoir mask, 8 L/min), respiratory rate was 24 breaths/min, blood pressure was 106/67 mmHg, heart rate was 101 beats/min, and body temperature was 37.2 °C. A chest radiograph taken at the time of admission is shown in Fig. 1A. The patient’s condition was stable with acceptable oxygenation level, and therefore, tracheal intubation was avoided, and the patient was treated with a high-flow nasal cannula (FIO_2_ of 60%, 50 L/min). From the time of ICU admission until 6 h later, the patient had no subjective symptoms, his respiratory rate was 22–30 breaths/min, SpO_2_ was stable at 92–98%, and there was no change in his consciousness. Transthoracic echocardiography (TTE) performed by a cardiologist confirmed that the cardiac function was within normal limits. However, 6 h after ICU admission, the patient suddenly began to complain of chills. His body temperature rose rapidly to 38.9 °C, and continued shivering was observed. The patient’s blood pressure increased rapidly along with shivering to 230/110 mmHg, his heart rate rose to 120 beats/min, and he became restless. Intravenous acetaminophen (1000 mg) was administered, followed by 50 mg of flurbiprofen (nonsteroidal anti-inflammatory drug [NSAID]); however, the symptoms remained unresolved. For high blood pressure, nicardipine (2–5 mg/dose) was administered; however, his blood pressure remained high, and his SpO_2_ began to decrease rapidly. The oxygen concentration of the high-flow nasal cannula was increased to 100%, but SpO_2_ decreased to 80%. Mechanical ventilation was necessary, and the patient’s trachea was intubated immediately.

Mechanical ventilation was initiated with the following settings: assist/control mode, pressure controlled, FIO_2_ at 100%, peak plateau pressure at 33 cmH_2_O, and positive end-expiratory pressure at 15 cmH_2_O. Immediately after intubation, blood gas analysis showed a PaO_2_ level of 38 mmHg indicating a rapid progression of hypoxia compared with that just before the start of shivering. Results of arterial blood gas analysis from the time of admission to the ICU until the introduction of ECMO is shown in Table 1. TTE showed no evidence of pulmonary embolism, such as right ventricular enlargement, and contractility mildly decreased to approximately 40–50% of the ejection fraction of the left ventricle. The patient was diagnosed with pulmonary edema due to afterload mismatch since other causes of hypoxia were ruled out, and a large amount of pink foam sputum was ejected from the endotracheal tube. The patient’s oxygenation did not improve despite continued ventilation for several hours, and the decision was made to introduce ECMO. A blood gas analysis immediately prior to the introduction of ECMO showed the PaO_2_ level at 42 mmHg (no change in ventilator settings) and a tidal volume of 280 mL (total respiratory system compliance, 15.5 mL/cmH_2_O). Chest radiographs obtained after starting ECMO are shown in Fig. 1B. Eleven days after starting ECMO, the patient was weaned from ECMO and placed on ventilatory management because of improvement in lung compliance. On day 26, a tracheostomy was performed, and the patient was weaned off the ventilator. The patient was discharged from the ICU on day 38. Seven days after discharge, the tracheostomy tube was removed, and 16 days later, the tracheostomy orifice was closed. The patient was discharged from the hospital 22 days after discharge from the ICU without any neurological sequelae.

**Table 1:** Results of arterial blood gas analysis from the time of admission to the ICU until the introduction of ECMO

	Admission to ICU	Pre-intubation	Post-intubation
FIO_2_	0.6	1.0	1.0
Setting	HFNC	HFNC	Intubated
pH	7.396	7.479	7.144
PaO_2_ (mmHg)	78.9	40.3	38.4
PaCO_2_ (mmHg)	43.3	31.0	77.0
SaO_2_ (%)	91.4	65.2	53.1
HCO3^−^ (mEq/L)	25.8	22.7	25.3
Base excess (mEq/L)	1.1	0.7	−7.0

## Discussion

We experienced a case in which hypoxia progressed rapidly and immediately after the start of shivering in a patient with COVID-19 who was in a stable condition and was managed with a high-flow nasal cannula. To our best knowledge, there are no case reports on hypoxia due to shivering requiring ECMO in a patient with COVID-19 or any other infectious disease.

It is controversial whether antipyretics should be given to such patients with COVID-19 who develop a fever. The febrile response activates the immune system through various pathways to suppress viral production and inactivation. In fact, a prospective, observational study showed that 28-day mortality increased in septic patients with fever who were administered NSAIDs to relieve the fever [4]. In contrast, fever activates the sympathetic nervous system, which increases the metabolic rate and disrupts the oxygen supply-demand balance [5]. When fever is accompanied by shivering, sympathetic nervous system activation can be synergistic, resulting in a rapid deterioration of cardiac function in patients without reserved cardiac function. Studies in patients with fever have shown that lowering body temperature increases stroke volume and cardiac output, suggesting that fever leads to a reduction in SVR [6].

Although the exact mechanism of hypoxia in our case is unclear, there is a possibility of three mechanisms. The first mechanism is the leakage of large amounts of fluid out of the pulmonary vessels due to afterload mismatch caused by a sudden increase in afterload due to shivering. In our case, there was a rapid rise in blood pressure immediately after shivering, and a large amount of pink frothy sputum was observed after intubation, suggesting that this mechanism may be the primary cause. Another possible mechanism is hypoxemia caused by increased oxygen consumption due to shivering; COVID-19 has a right-left shunt due to a small intravascular pulmonary thrombus. Under such conditions, when oxygen consumption increases and SvO2 decreases, arterial blood oxygen saturation becomes low even without an increase in shunt blood flow because of the low oxygen saturation of the shunt blood flow. In addition, an increase in heart rate due to shivering generally increases pulmonary blood flow velocity. Normally, the time it takes for blood to pass through the lungs is 0.7 s, which is well greater than the 0.2 s of oxygen diffusion time. Therefore, little blood is perfused into the left atrium without being oxygenated. However, if diffusion capacity is impaired and pulmonary blood flow velocity is increased, the time for blood to pass through the lungs is shorter, resulting in hypoxemia. However, since this mechanism of decreased oxygenation requires normal cardiac contractility, it is unlikely to be as pronounced when contractility is reduced as in this case. However, either mechanism can occur following shivering, attention should be paid to the patient’s oxygenation when shivering occurs.

Other conditions that should be differentiated include rapid deterioration of COVID-19. The primary mechanism of hypoxia in COVID-19 shifts chronologically to neutrophil-induced inflammation, as in other acute respiratory distress syndromes [2]. However, these changes usually progress on a daily basis, and no case has been reported to progress rapidly, making it unlikely.

In conclusion, we experienced a case of COVID-19 in which the patient’s oxygenation deteriorated, requiring ECMO. The mechanism of hypoxia was speculated to be an afterload mismatch triggered by shivering. Extreme caution should be given to the hemodynamic state in these conditions.

## Abbreviations

ICU: Intensive care unit
ECMO: Extracorporeal membrane oxygenation
FIO_2_: Fraction of inspired oxygen
NSAIDs: Nonsteroidal anti-inflammatory drugs
SAT: Spontaneous awakening test
SVR: Systemic vascular resistance
PaCO_2_: Partial pressure of carbon dioxide
PaO_2_: Partial pressure of oxygen
COVID-19: Coronavirus disease 2019
SpO_2_: Oxygen saturation
CT: Computed tomography
